# Outlining the Ancestry Landscape of Colombian Admixed Populations

**DOI:** 10.1371/journal.pone.0164414

**Published:** 2016-10-13

**Authors:** Humberto Ossa, Juliana Aquino, Rui Pereira, Adriana Ibarra, Rafael H Ossa, Luz Adriana Pérez, Juan David Granda, Maria Claudia Lattig, Helena Groot, Elizeu Fagundes de Carvalho, Leonor Gusmão

**Affiliations:** 1 Pontificia Universidad Javeriana, Facultad de Ciencias, Bogotá, Colombia; 2 Laboratório de Genética y Biología Molecular, Bogotá, Colombia; 3 DNA Diagnostic Laboratory (LDD), State University of Rio de Janeiro (UERJ), Rio de Janeiro, Brazil; 4 i3S (Instituto de Investigação e Inovação em Saúde), Universidade do Porto, Porto, Portugal; 5 IPATIMUP (Instituto de Patologia e Imunologia Molecular da Universidade do Porto), Porto, Portugal; 6 IdentiGEN - Genetic Identification Laboratory and Research Group of Genetic Identification, Institute of Biology, School of Natural and Exact Sciences (FCEN), University of Antioquia, Medellin, Antioquia, Colombia; 7 Universidad El Bosque, Facultad de Medicina, Bogotá, Colombia; 8 Laboratorio de genética humana, Universidad de los Andes, Bogotá, Colombia; National Cheng Kung University, TAIWAN

## Abstract

The ancestry of the Colombian population comprises a large number of well differentiated Native communities belonging to diverse linguistic groups. In the late fifteenth century, a process of admixture was initiated with the arrival of the Europeans, and several years later, Africans also became part of the Colombian population. Therefore, the genepool of the current Colombian population results from the admixture of Native Americans, Europeans and Africans. This admixture occurred differently in each region of the country, producing a clearly stratified population. Considering the importance of population substructure in both clinical and forensic genetics, we sought to investigate and compare patterns of genetic ancestry in Colombia by studying samples from Native and non-Native populations living in its 5 continental regions: the Andes, Caribe, Amazonia, Orinoquía, and Pacific regions. For this purpose, 46 AIM-Indels were genotyped in 761 non-related individuals from current populations. Previously published genotype data from 214 Colombian Natives from five communities were used for population comparisons. Significant differences were observed between Native and non-Native populations, among non-Native populations from different regions and among Native populations from different ethnic groups. The Pacific was the region with the highest African ancestry, Amazonia harboured the highest Native ancestry and the Andean and Orinoquían regions showed the highest proportion of European ancestry. The Andean region was further sub-divided into 6 sub-regions: North East, Central West, Central East, West, South West and South East. Among these regions, the South West region showed a significantly lower European admixture than the other regions. Hardy-Weinberg equilibrium and variance values of ancestry among individuals within populations showed a potential stratification of the Pacific population.

## Introduction

In a geographic framework, Colombia occupies the southern extreme of the bridge that connects the two American subcontinents. Therefore, since prehistorical times, Colombia has been subject to an intense genetic and cultural flow carried by Native American migrations, which ultimately resulted in a high diversity of ethnic groups inhabiting the country and to a noticeable heterogeneity between geographic regions [1,2] (DANE censo general 2005; http://www.dane.gov.co). This heterogeneity is still maintained in the current Colombian population, whose diversity has been further shaped by admixture with people from other continents. The Andes is the region in which the Chibcha civilization flourished, the third most developed group in the Americas after the Aztecs and the Incas. The Caribbean region is clearly differentiated from the remaining regions, and its territory is inhabited by highly diverse ethnic groups. These two regions, both before and after the European conquest, were and remain the most densely populated and economically active in the country. The other three natural regions include the Pacific region and the forest area that today corresponds to the Orinoquía and Amazonia regions, which are the largest in the territory but the least populated and least economically developed (DANE censo general 2005).

During and after the colonial era, the genetic background of the populations currently inhabiting the Colombian territory was ultimately shaped by different levels of admixture between Natives and European and African incomers [3–5]. This admixture was associated with different migration patterns, drift effects and the more or less pronounced geographic isolation of the populations.

Some studies have been conducted to genetically characterize Colombian populations and to correlate the observed diversity with history events, using genetic markers with different inheritance patterns and degrees of susceptibility to detect populations’ differentiation by mutation, drift, and admixture [6–10].

The genetic ancestry of Colombia has been investigated using lineage markers. Several studies were published describing the Y chromosome profile of Colombian populations, but most of these studies were restricted to Y-STRs (e.g., [11–13]), and only a few included the Y-SNP markers that allow for a more robust analysis of the paternal ancestry of a population [7,10,14–16]. Some of the above-mentioned studies also included mtDNA data, revealing the unequal maternal *vs*. paternal ancestry of the admixed Colombian populations, which harbour a gene pool that is mainly composed of Native American mtDNA and European Y chromosome haplogroups [7,14–16]. European mtDNA haplogroups are the second-most represented in non-Native populations in Colombia, with the exception of some African descendant populations in which the African L-haplotypes are predominant [6,17,18]. Most Native groups still preserve an almost complete native maternal ancestry, and signs of European admixture can be observed in the Y chromosome gene pool that varies with the degree of cultural and geographic isolation of the group [16,19,20].

Although uniparental markers have been useful in revealing differences in populations with respect to paternal and maternal inheritance, a comprehensive description of the genetic profile of populations in terms of ancestry can only be achieved by the study of recombining markers.

Regarding recombining autosomal markers, the studies available for Colombia have been even more fragmentary than for lineage markers and have only considered a restricted number of markers and/or population groups (e.g., [3,7,15,21]). Price et al. [22] have studied a large set of ancestry-informative markers (AIMs); however, in this study, the “Colombians” were represented by individuals from a single population (Antioquia), and did not account for the large heterogeneity in the country. A much larger number of population samples from across Colombia were examined by Rojas et al. [7] and Ibarra et al. [8]. However, the study by Rojas et al. [7] included only a small number of eleven autosomal AIMs, and the set of 52 SNPs studied by Ibarra et al. [8] does not present high levels of intercontinental diversity. Therefore, although appropriate for estimating relative differences in the ancestral composition of the populations analysed, the data from both studies are not sufficient to obtain accurate estimates of the ancestry of Colombians. So far, these studies have shown that Colombia does not contain a uniform genetic pool; rather, it has a high heterogeneity of African, European and Native American ancestries, depending on the region of the country.

Considering the complex history and the high genetic heterogeneity of the present-day populations in Colombia, more comprehensive studies are still required for a fine-scale mapping of the admixture structure of the country, which is crucial in many applied fields, including clinical and forensic genetics [23,24].

In an attempt to contribute to a better understanding of the ancestry of Colombians, in the present work we used a panel of 46 autosomal ancestry-informative insertion/deletion markers (AIM-Indels) to characterize patterns of variation across Native and non-Native Colombian populations from different geographic regions.

The AIM-Indels that we used were previously selected by Pereira et al. [25] for inference of ancestry and admixture proportions of African, European, Native American and East Asian origin, and they have been applied to the study of South American populations from Bolivia, Brazil and Colombia [16,26,27].

Genetic variation in the Native and non-Native populations occupying the five continental regions following the settlement of Colombia (the Andean, Caribbean, Pacific, Orinoquía, and Amazonia regions) was investigated with an emphasis on the Andean region, which currently represents almost 80% of the total population (DANE censo general 2005). The results revealed a highly stratified population in terms of ancestry that should be considered when delineating studies and/or interpreting genetic data in different areas of research.

## Materials and Methods

### Ethics Statement

All samples involved in the study were anonymised DNA extracts that had been previously obtained from healthy, unrelated individuals. Blood samples were collected by free and written informed consent for research purposes. The current study complies with the ethical principles of the 2000 Helsinki Declaration of the World Medical Association and, together with the informed consent, it was approved by the Ethics Committee of the Pontificia Universidad Javeriana of Colombia.

### Sample collection and DNA extraction

**Fig 1:**
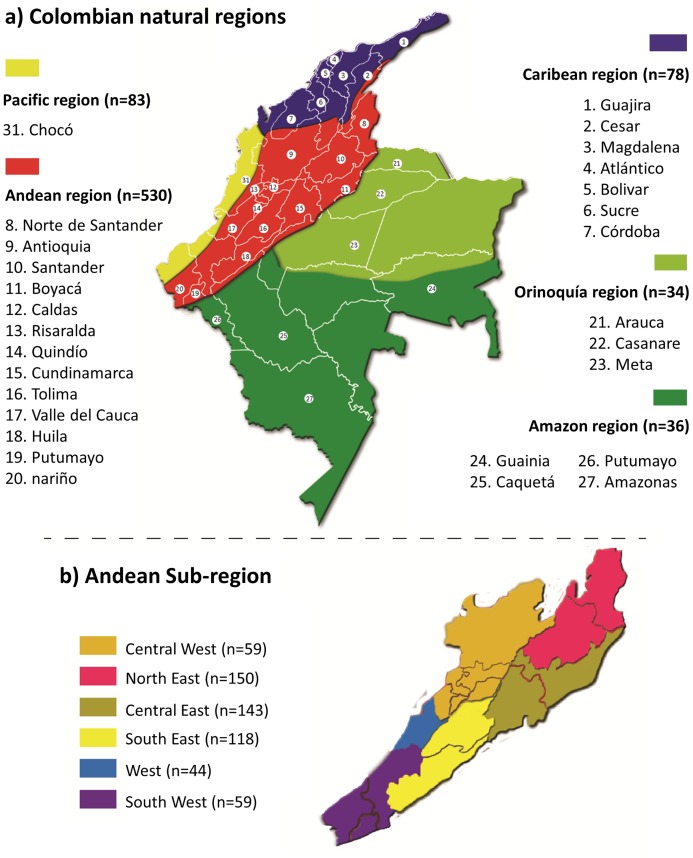
Map of the continental territory of Columbia showing the five main regions and geographical locations of sampling sites (a). The Andean sub-regions considered in the present work and their sample sizes are also indicated (b).

A total of 761 samples were collected from unrelated individuals from 28 of the 32 departments of Colombia, as indicated in Fig 1a. These are random samples representing the general population of each region, comprising individuals with admixed ancestry (Afro-Colombians and Mestizos), mainly from urban centres in each department. In the present manuscript, the term non-Native was used to distinguish these populations from the Indigenous tribes described in previous studies, which were used for comparison.

A larger sample was collected from Departments in the Andes because this is the most densely populated region of Colombia. This region was further subdivided into 6 sub-regions. The location and number of samples included in this study for each Natural Region of Colombia and for the Andean sub-regions are indicated in Fig 1.

DNA was extracted following the salting-out protocols described by Miller et al. [28]. DNA quantification was performed by spectrophotometry using the Thermo Scientific NanoDrop 100 apparatus (NanoDrop Technologies, Wilmington, DE, USA). Aliquots of DNA were prepared for PCR reactions at a concentration of 2.5 ng/μL.

For analysis, we included data from Native Colombian populations that were previously studied for the same AIM-Indel markers. These included (i) Natives living in the Cauca region; (ii) Emberá-Chami living in the Antioquia Department; (iii) Natives living in Guainía; (iv) Motilón-Barí living in Norte de Santander; (v) and Pijao from the Tolima Department [16,29]. These Native groups are from different departments (see Fig 1 in Ossa et al. [29]) and belong to diverse linguistic/ethnic groups: Barbacoan (Guambiano and Coconuco from Cauca), Chibchan (Motilón-Barí; Nasa from Cauca), Chocoan (Emberá-Chamí), Tucanoan (Tucano, Cubeo, Guanano and Desana from Guanía) and Arawakan (Curripaco from Guainía). Additionally, to perform ancestry analysis, we used reference data available for HGDP-CEPH samples from African, European and Native American populations [25].

### Genetic markers and genotyping

All samples were genotyped for 46 AIM-Indel markers distributed across the autosomal genome. PCR reactions were carried out in a single multiplex containing all 46 primer pairs according to the protocol described by Pereira et al. [25] after adjusting the total reaction volume to 5 μL for 2.5 ng of template DNA. The following PCR thermocycling conditions were used: an initial step of 15 min at 95°C, followed by 27 cycles at 94°C for 30 s, 60°C for 1.5 min, 72°C for 45 s and a final extension at 72°C for 60 min.

Dye-labelled PCR amplified fragments were separated by capillary electrophoresis and detected using an Applied Biosystems^®^ 3500 Series Genetic Analyzer (Thermo Fisher Scientific Inc., Waltham, Massachusetts, USA). Automated allele calls were obtained with GeneMapper v.4.1 software (Thermo Fisher Scientific, Inc.).

### Statistical analyses

Genetic diversity parameters, including the estimation of allele frequency, observed and expected heterozygosity, Hardy-Weinberg values, *F*_ST_ genetic distances and resulting non-differentiation *p*-values, were assessed using Arlequin v3.5 [30]. Using this software, the significance of genetic distances is obtained by permuting individuals between populations; and the *p*-value of the test is the proportion of permutations leading to a *F*_ST_ value larger or equal to the observed one. A multidimensional scaling (MDS) plot of the pairwise *F*_ST_ matrix was created using the software STATISTICA v7.0. (Statsoft, Tulsa, Oklahoma; http://www.statsoft.com/).

The apportionment of genetic ancestral contributions to the different regions of Colombia was estimated as the mean of each ancestry across individuals, using Admixture v1.3 software [31]. As suggested by cross validation results of a preliminary unsupervised analysis, and considering the historical formation of the Colombian population, we assumed an essentially tri-hybrid contribution from Native Americans, Europeans and Africans (i.e., K = 3). To estimate the ancestral membership proportions in the studied populations, supervised analyses were then performed using prior information on the geographic origin of the reference samples. Ancestry analyses were initially conducted using the HGDP-CEPH populations as a reference [25]. We then investigated the possible impact of considering different Native groups (i.e., HGDP-CEPH, Emberá-Chami, Motilón-Barí and Guainía) as the American ancestral contributor when estimating genetic ancestry. Based on the coherence of the results (see the discussion below), we conducted a final supervised analysis in which the American reference comprised all samples from those four Native groups that showed individual ancestry estimates ≥ 90% Native in the preliminary unsupervised analysis.

## Results and Discussion

The genotyping results obtained for the 761 Colombian samples from the present work and the 121 samples from the Native groups examined in Ossa et al. [29] are listed in S1 Table. The genotypes from the remaining 93 samples are available in Xavier et al. [16]. Based on the observed genotypes, allele frequencies were estimated in each population and are presented in S2 Table. Hardy-Weinberg tests showed no significant deviations in all population samples studied, with the exception of MD1636 in the Pacific region (S3 Table). A significance level of 0.001 was obtained by applying Bonferroni’s correction to 46 tests performed in each population sample. It is worth mentioning that *p*-values below 0.05 were also observed for six additional markers in the Pacific population sample; in most cases (six out of seven), these markers were associated with an excess of homozygotes.

### Ancestry analysis among the five natural regions of continental Colombia

Based on geographical criteria, Colombia can be divided into six natural regions, with the three Andes Mountain branches (East, Central and West Andes) separating the two coastal regions (Pacific and Caribe) from the two inner regions (Orinoquía and Amazonia). A sixth region includes the islands in both the Caribbean Sea and the Pacific Ocean (Insular region).

**Fig 2:**
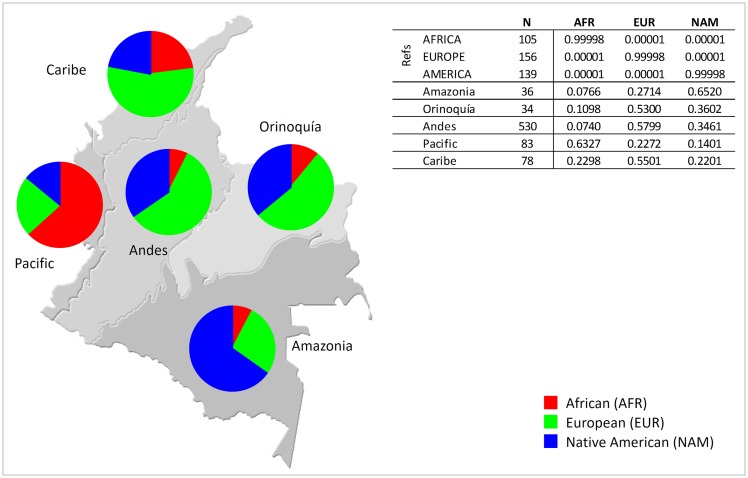
African, European and Native American membership proportions in samples from the five continental regions of Colombia.

The apportionment of ancestral genetic contributions from Africa, Europe, and Native America were estimated in five continental regions of Colombia: Caribe, Andes, Orinoquía, Amazonia and Pacific (Fig 2). The prevalent ancestral component in Caribe, Andes and Orinoquía was the European; the Pacific and Amazonia regions showed a higher genetic contribution from Africa and Native America, respectively.

The Andes region had the highest European contribution, but also revealed a marked Native American contribution. Together with Amazonia, this region had the lowest values of African ancestry. A similar genetic ancestry profile was observed in Orinoquía, although with slightly higher Native American and African components than in the Andean region.

The high European contribution in Andean populations is in accordance with historical data because, during European colonization, the Magdalena River, located between the Central and Western branches of the Andes, was the main route of dispersal through the interior of the territory.

Amazonia had low values of both European and African gene flow, while more than 65% of its genes are Native American. This is probably due to the geographic isolation of this region, which has the lowest population density in the country. Although it had a higher proportion of non-Native influx than some Native groups (previously studied by Xavier et al. [16] and Ossa et al. [29] for the same markers), the sample from Amazonia showed a similar proportion of Native American ancestry as the Pijao group from Tolima [29].

The Pijao (also known as Coyaima-Natagaima) originate in different ethnic groups that share linguistic and cultural affinities. They lost their original language, and their culture and religion experienced some degree of acculturation with Hispanic influence [32].

The similarity in the proportion of Native ancestry found in the Pijao and the population from Amazonia highlights the importance of geographic isolation in the preservation of high levels of Native ancestry in non-Native communities. It also emphasizes that the self-perception of belonging to an ethnic group cannot be determined by genetics alone, as it is the case of the Pijao indigenous community in Colombia that although subjected to a high European admixture still preserves Native American ancient traditions.

As expected from its historical African background, the sample from the Pacific region has 63% African ancestry and the lowest values of both European and Native American ancestries of all regions in the country. After the arrival of the Europeans, the Pacific coast suffered different migration waves that changed its original Native American genepool. During the 16^th^ and 17^th^ centuries, a large number of African slaves were brought to work in gold mines and, during this period, the African population become predominant. This population pattern was reinforced with the abolition of slavery in the 19^th^ century, when a large number of Africans came to this region, especially from the Department of Cauca.

Although much lower than in the Pacific, the Caribe region also presents a significant African ancestry (23%), which can be explained by the role of Cartagena as one of the most important ports in South America during the slave trade.

### Ancestry analysis among Andean sub-regions

**Fig 3:**
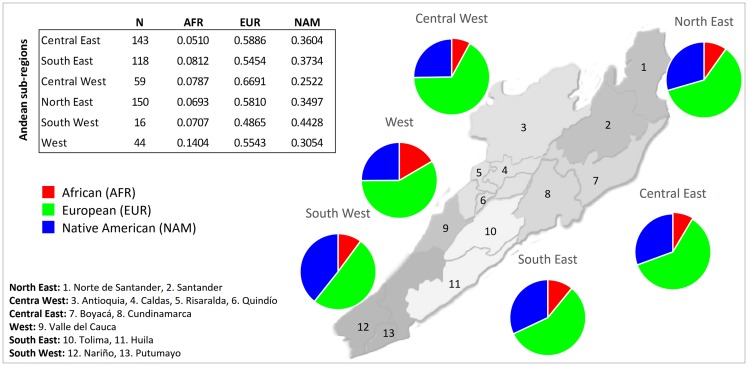
African, European and Native American membership proportions in samples from the Andean sub-regions of Colombia.

The Andes is the most densely populated region of Colombia. Based on cultural criteria, this region was divided into six sub-regions: Central West, West, South West, North East, Central East, and South East. When comparing the ancestry of populations across the Andes, all sub-regions are characterized by a predominant European contribution (Fig 3). Native Americans represent the second-highest contribution to the genetic background of these populations. Among the Andean sub-regions, the Central West revealed the highest European and the lowest Native American contributions (67% and 25%, respectively). This sub-region is also known as the Antioqueña colonization region because the first Europeans were established in the Antioquia Department and subsequently migrated to the neighbouring departments of Caldas, Risaralda, Quindío, Norte del Valle and Norte del Tolima.

The highest African contribution (14%) is found in the West sub-region (also known as Valle del Cauca), which is the Andean region that is closest to the Pacific coast. The two southern regions, including populations from the Departments of Tolima, Huila (South East), Nariño, Cauca and Putumayo (South West), have preserved a marked Native American ancestry (37–44%).

The North East (comprising the Norte de Santander and Santander Departments) and Central East (Cundinamarca and Boyacá) sub-regions presented a similar ancestry profile and, although these two Andean sub-regions had been early colonized by Europeans that largely displaced the Native populations, their samples exhibited lower European ancestry than those from the Central West region.

### Comparison with previous studies

In this study, we obtained higher differences among European, African and Native American contributions than Ibarra et al. [8] due to the improved suitability of the markers selected for ancestry estimation (S4 Table). In fact, when markers with low levels of population differentiation are used to infer contributions from three source populations, they tend to overestimate values of ancestry below one third and underestimate contributions that are above this value [33]. Despite the differences observed in the absolute values of ancestry, the relative values among regions are similar in both studies: Orinoquía and most Andean populations showed higher European ancestry, followed by Native ancestry, and the Pacific populations showed a predominantly African ancestry. Differences were observed between the two samples from the South East region (S4 Table) that cannot be attributed to differences in the type of marker used. The sample from Ibarra et al. [8] (comprising individuals from Nariño) has a higher Native American contribution than that studied in this work (comprising individuals from Nariño and Putumayo). This result can be attributed to a sampling effect or may be caused by differences between Nariño and Putumayo populations. Regardless, it would be desirable to perform a larger study covering the three departments in this region (Cauca, Nariño and Putumayo) to investigate their population substructure. The need for such an analysis is further emphasized by the high intra-population variation that was observed during this study.

The results we obtained are more difficult to reconcile with those reported by Rojas et al. [7] for eleven autosomal AIMs. In general, we obtained higher estimates of European and African ancestry in Andean populations and lower estimates of African contribution in Caribbean and Valle de Cauca (West Andean) populations. Additionally, differences among Andean sub-regions were lower in our study.

In previous studies, the ancestry of four Native groups from the Andes was investigated using the same set of AIM-Indel markers employed in this work [16,29]. When comparing the non-Native admixture, no correlations were observed between the Native and non-Native groups of each sub-Andean region. For instance, the Natives from Cauca (in the South West sub-region) and the Pijao (in the South East sub-region) both present high levels of admixture with non-Native gene pools. Conversely, although from geographic sub-regions with higher admixture with Europeans, the Emberá-Chamí (Central West) and the Motilón-Barí (North East) reveal the highest values of Native ancestry, highlighting the importance of cultural and geographic isolation in the preservation of the Native genetic heritage of these Andean Native groups.

### Inter-individual ancestry variation within populations

**Fig 4:**
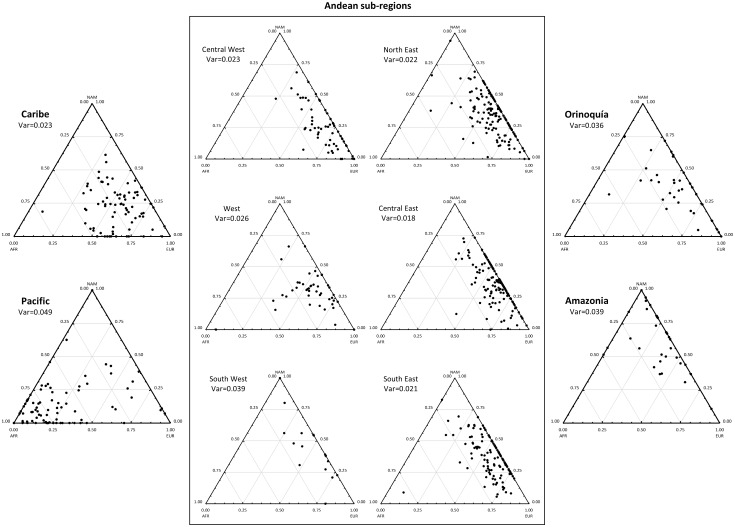
Individual ancestry estimates within populations obtained in samples from the five regions of Colombia. The average value of variance for the three estimates in each population (var) are also indicated.

To investigate the signals of recent admixtures or population stratifications, we compared the variations in individual ancestry estimates within the studied populations. Therefore, African, European and Native American membership proportions were assessed using the Admixture software, and the results are presented in Fig 4. A high inter-individual variation can be observed within populations, especially in the Pacific, Orinoquía, Amazonia and South West Andean regions. These populations showed higher values of average variance of African, European and Native American ancestries than the remaining populations from the Andes region and the Caribbean region (Fig 4). In S1 Fig we plotted the absolute values obtained after subtracting the individual ancestry estimates from the average value of the population. Confirming our previous observations, most individuals from the Andean (except the South West sub-region) and Caribbean populations have ancestry values that are closer to the population average, in contrast to the South West and Pacific regions, where a high proportion of the individuals show ancestry values far from the average.

The high intra-population variation found in the Pacific regions can be the result of (i) recent migration and admixture between two groups that differ in their African ancestry; (ii) the presence of population substructure in an old admixed population; or (iii) both. A recent migration of Africans is known to have occurred to the North Pacific region after the abolition of slavery. However, the new incomers joined a population that was already known to harbour an important African component. Therefore, a certain degree of population substructure can be expected in the Pacific population.

In conclusion, regions with less European admixture showed higher ancestry variation among individuals, indicating more recent admixture events and/or a stronger stratification of these populations.

### Genetic distance among Colombian Native and non-Native populations

Pairwise *F*_*ST*_ genetic distances were calculated between Native and non-Native populations from Colombia and three reference samples from Africa, America and Europe.

As expected, higher values of genetic distance were obtained for the AIM-Indels included in the present work than for the SNPs previously studied by Ibarra et al. [8], which have been selected to maximize intra-population differentiation.

**Fig 5:**
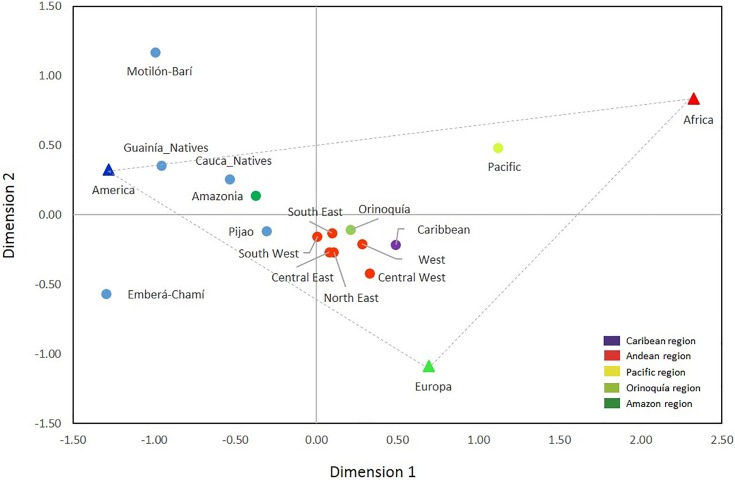
MDS plot of the *F*_*ST*_ pairwise genetic distances between Native and non-Native Colombian populations and the three samples used as references for Africa, Europe and Native America (stress = 0.057136).

Significant differences were observed between Native and non-Native populations, as well as among non-Native populations from different regions and among native populations from different ethnic groups (S5 Table). When comparing the Andean sub-populations, no significant differences were found except between the Central West and North and South East. The presence of the highest European ancestry in the Central West can explain these results, although differences between the Native backgrounds of these Andean sub-populations may also exist. Indeed, significant differences were found between Natives, even for groups with similar membership proportions in ancestry estimates, including the Emberá-Chamí (Central West) and the Motilón-Barí (North East). The large genetic distance between these two Native groups and between them and the HGPD-CEPH reference sample from Native Americans reveals important genetic drift events, that are visualized in the MDS plot in Fig 5.

In the MDS plot, the Pacific sample appears closer to the African reference sample, the Amazonia sample is closer to the American Native sample, and the Andean, Orinoquía and Caribbean samples are closer to the European reference sample (Fig 5).

In summary, the position of the non-Native populations in the MDS plot closely follows the expected results based on the ancestry estimates. The high genetic distances observed between Native populations with low non-Native American influx (HGDP-CEPH reference samples from Native American, Guainía, Emberá-Chamí and Motilón-Barí groups) cannot be explained solely by differences in the ancient background of these populations; these populations must have been subject to a strong genetic drift, including founder and/or endogamy events.

Genetic differences among nearby Native American groups have been previously reported based on different types of markers, namely mtDNA, Y chromosome, HLA class II variation and ancestry informative SNPs [10,16,34–36]. The results obtained point to a subpopulation differentiation process, due to a restricted gene flow with the Andes mountains acting as a geographic barrier, or as the result of different migration routes entering South America, from Panama, during the Pleistocene [3,21,37].

### The impact of Native American reference samples in ancestry estimates

Methods of inference of genetic ancestry usually model hypothetical ancestral populations or rely on the use of samples representing those putative ancestral contributors. This modelling is not always a straightforward task because, for most genetic studies, only contemporary populations are available to be used as a reference. Such populations may harbour a more or less preserved ancestral genepool. Concerning the populations under study, it is believed that since the first admixture events with Europeans or Africans, an important part of Native American genetic diversity has been gradually lost. This loss of diversity has been caused by genetic drift, creating noticeable genetic distances among groups.

Therefore, considering the perceptible differentiation among the Native American groups discussed above, we further assessed its putative impact in the inference of genetic ancestry when, for instance, using different groups as the Native American reference population. S6 Table summaries ancestry analyses obtained for the same dataset while considering HGDP-CEPH and each of the Colombian Native groups as the reference population (i.e., Emberá-Chamí, Guainía and Motilón-Barí). The results were remarkably similar, with only small variations in the ancestry values obtained for the Colombian populations under study. This observation supports the overall robustness of the ancestry analyses performed, which seem to adequately represent the genetic diversity existing among Native American groups.

### Conclusion

This study provides a general picture of the ancestry of the Colombian populations from the five continental regions of the country, complementing previous information on lineage and recombining markers. The overall results revealed a highly stratified population in terms of ancestry both between and within delimitated natural regions. This stratification should be taken into account when delineating studies and/or interpreting genetic data in different areas of research.

## Supporting Information

S1 Fig Plot of the absolute values obtained after subtracting the individual ancestry estimates from the average value of the population. Each individual is represented along the x-axis, by three points corresponding to the differences in African, European and Native American ancestries to the average.
(TIF)

S1 Table List of 46 AIM-Indel genotypes from the Colombian samples included in the present study and in Ossa et al. (2015). (XLSX)

S2 Table Frequencies of the shorter alleles (allele 1) for the 46 AIM-Indels in Colombian populations. For loci with 3 alleles in a population sample, the frequency of a second allele (allele 2) was also indicated.
(XLSX)

S3 Table Observed and expected heterozygote values and p-value for the exact test of Hardy-Weinberg equilibrium (forecasted chain length: 1,000,000; dememorization steps: 100,000), excluding the monomorphic loci (*). *p*-values above 0.05 are indicated in pink, and those below 0.001 are indicated in yellow.
(XLSX)

S4 Table Comparison of the African, European and Native American ancestry estimates obtained in this study and in Ibarra et al. (2014). (XLSX)

S5 Table Pairwise *F*_ST_ values between Colombian and reference populations (below diagonal) and corresponding differentiation p-values (above diagonal; significant values after Bonferroni’s correction [*p*<0.0005] are indicated in red). (XLSX)

S6 Table Comparative ancestry estimates of Colombian populations considering different Native American groups as the ancestral reference in supervised analyses. (XLSX)
