# Supplementary figures and images for: Outlining the Ancestry Landscape of Colombian Admixed Populations
Source: PLoS One. 2016 Oct 13;11(10):e0164414. doi: 10.1371/journal.pone.0164414 (PMC5063461; doi:10.1371/journal.pone.0164414)

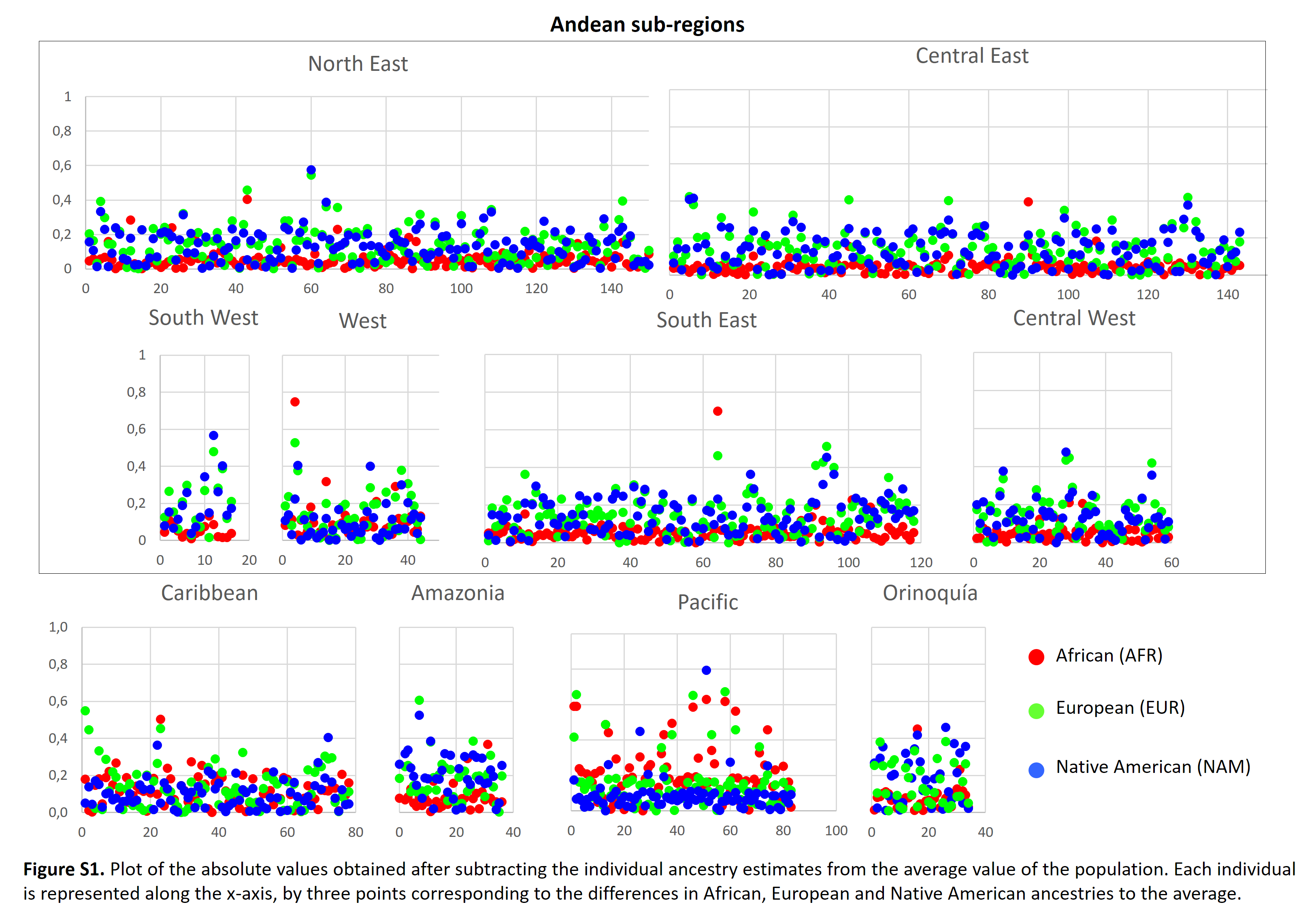

Supplement: S1 Fig — Each individual is represented along the x-axis, by three points corresponding to the differences in African, European and Native American ancestries to the average. (TIF) [file pone.0164414.s001.tif]
